# Plasma proteome-wide Mendelian randomization reveals the association of extracellular matrix proteins with abdominal aortic aneurysm

**DOI:** 10.1016/j.jvssci.2025.100290

**Published:** 2025-05-08

**Authors:** Samuel Khodursky, Shuai Yuan, Joshua M. Spin, Philip S. Tsao, Michael G. Levin, Scott M. Damrauer

**Affiliations:** a Department of Surgery, University of Pennsylvania Perelman School of Medicine, Philadelphia, PA; b Corporal Michael J. Crescenz VA Medical Center, Philadelphia, PA; c Unit of Cardiovascular and Nutritional Epidemiology, Institute of Environmental Medicine, Karolinska Institutet, Stockholm, Sweden; d VA Palo Alto Healthcare System, Palo Alto, CA; e Department of Medicine, Stanford University School of Medicine, Stanford, CA; f Stanford Cardiovascular Institute, Stanford University School of Medicine, Stanford, CA; g Division of Cardiovascular Medicine, Department of Medicine, University of Pennsylvania, Perelman School of Medicine, Philadelphia, PA; h Department of Genetics, Perelman School of Medicine at the University of Pennsylvania, Philadelphia, PA

**Keywords:** AAA, Mendelian randomization, ECM, LTBP4, COL6A3

## Abstract

**Objective:**

Abdominal aortic aneurysm (AAA) is a common and life-threatening vascular disease. Genetic studies have identified numerous risk loci, many potentially encoding plasma proteins. However, the causal effects of plasma proteins on AAAs have not been thoroughly studied. We used genetic causal inference approaches to identify plasma proteins that have a potential causal impact on AAAs.

**Methods:**

Causal inference was performed using two-sample Mendelian randomization (MR). For AAAs, we utilized recently published summary statistics from a multi-population genome-wide association meta-analysis including 39,221 individuals with and 1,086,107 individuals without AAAs from 14 cohorts. We used protein quantitative trait loci (protein quantitative trait loci) identified in two large-scale plasma-proteomics studies (deCODE and UKB-PPP) to generate genetic instruments. We tested 2783 plasma proteins for possible causal effects on AAAs using two-sample MR with inverse variance weighting with common sensitivity analyses.

**Results:**

MR identified 90 plasma proteins associated with AAAs at a false discovery rate <0.05, with 25 supported by colocalization analysis. Among those supported by both MR and colocalization were proteins such as PCSK9 (odds ratio [OR], 1.3; 95% confidence interval [CI], 1.2-1.4; *P* < 1e-10), LTBP4 (OR, 3.4; 95% CI, 2.6-4.6; *P* < 1e-10), and COL6A3 (OR, 0.6; 95% CI, 0.5-0.7; *P* < 1e-6). Gene Ontology analysis revealed enrichment of proteins (extracellular matrix; OR, 7.8; *P* < 1e-4), some with maximal mRNA levels in aortic tissue. Bi-directional MR suggested plasma level changes were not caused by liability to AAA itself. Colocalization analysis showed that an aortic expression quantitative trait locus for COL6A3, and a splicing quantitative trait locus for LTBP4 colocalized with their respective plasma pQTLs and AAA signals.

**Conclusions:**

Our results highlight proteins and pathways with potential causal effects on AAAs, providing a foundation for future functional experiments. These findings suggest a possible causal pathway whereby genetic variation affecting extracellular matrix proteins expressed in the aortic wall cause their levels to change in blood plasma, influencing development of AAAs.

## Clinical Relevance

Here, we used plasma proteome-wide Mendelian randomization to identify 90 proteins with possible causal associations with abdominal aortic aneurysms. We found that these proteins were enriched for proteins typically found in extracellular matrix and the aorta. Finally, we linked aortic expression changes in LTBP4 and COL6A3 to changes in their plasma levels, and increased liability for abdominal aortic aneurysms. These findings lay the groundwork for potential clinical applications, including new therapeutic targets and blood-based biomarker screening tests.

**Article Highlights**

• **Type of Research:** Human study
• **Key Findings:** We identified 90 plasma proteins associated with abdominal aortic aneurysms (AAAs) using Mendelian randomization. This set was enriched for proteins typically found in the extracellular matrix (ECM) and the aortic wall. Additionally, we found an association between changes in the aortic expression of two of these ECM proteins (LTBP4 and COL6A3), their plasma levels, and liability for AAAs.
• **Take Home Message:** These findings suggest a possible causal pathway whereby genetic variation affecting ECM proteins expressed in the aortic wall cause their levels to change in blood plasma, influencing development of AAAs.

Abdominal aortic aneurysm (AAA) is a relatively common and life-threatening vascular disease characterized by the expansion of the infrarenal aorta. This expansion can lead to rupture, resulting in over 40,000 deaths annually in the United States.^1^ Despite this burden, there are no pharmacological therapies that are currently approved to treat AAAs.^2^

Multiple genetic studies have identified risk loci associated with AAA susceptibility, with a recent genome-wide association study (GWAS) meta-analysis identifying over 100 independent loci.^3^, ^4^, ^5^, ^6^, ^7^ These studies, and others, have shown that AAA is a complex disease likely caused by a variety of interacting factors including inflammation and oxidative stress, along with extracellular matrix (ECM) and lipid metabolism dysfunction.^8^ Additionally, many of the loci associated with AAAs encode proteins found in blood plasma.^3^

Observational studies are limited by their inability to establish causality, as they can only identify correlations between genetic variants and traits without controlling for confounding variables. Furthermore, observational studies typically cannot infer the direction of potential causal relationships: for example, they do not distinguish whether changes in expression of a given protein cause a phenotype or are caused by the phenotype. Mendelian randomization (MR) is a statistical technique that utilizes genetic variation to infer potentially causal relationships between exposures and outcomes.^9^ In a manner analogous to randomization in a randomized control trial, MR leverages the random assortment of genetic variants at conception, as described by Mendel’s laws of inheritance, to help circumvent issues of confounding and reverse causation that can affect observational studies. By identifying genetic variants associated with an exposure of interest, MR can assess whether those variants are also associated with an outcome, allowing one to measure the potential causal effect of the exposure on the outcome. This approach has been successfully used to identify therapeutic targets for disease.^10^^,^^11^ Importantly, drugs supported by genetic evidence (such as MR) are 2 to 3 times more likely to be approved.^12^

Recent advances have allowed for the quantification of thousands of plasma proteins and the identification of genetic variants associated with their levels (protein quantitative trait loci [pQTLs]).^13^^,^^14^ These methods rely on either chemically modified nucleotide aptamers (eg, SomaScan) or antibodies (eg, Olink) to quantify the levels of thousands of proteins found in the plasma of individuals. Under certain assumptions, MR allows one to use this data to assess the putative causal effect of plasma protein levels on AAAs. Given the proteome-wide nature of this data, MR can be utilized as a high-throughput computational screen to identify possible therapeutic targets.

In the framework of MR, we treated plasma protein levels as the exposures and AAAs as the outcome. More specifically, we used pQTLs identified in two large-scale plasma-proteomics studies to generate instrumental variables for plasma protein levels.^13^^,^^14^ We then combined this with summary statistics from a recent GWAS meta-analysis for AAAs^3^ to run a plasma-proteome-wide MR screen to identify blood proteins with putative causal effects on the formation of AAAs.

## Methods

### Computation and statistical analysis

All analyses were performed in ‘R’ version 4.3.2. We used the ‘R’ package ‘targets’ (version 1.7.1) to create a pipeline.^15^

### Initial MR analysis

Causal inference was performed using two-sample MR. The three core assumptions underlying MR are: (1) the genetic variants used as instrumental variables (IVs) are associated with the exposure; (2) the IVs are only associated with the outcome through the exposure (and not through another factor); and (3) the IVs are not associated with any confounders that influence the exposure and the outcome.^10^^,^^16^ In practice, the first assumption can be met by using variants that are significantly associated with the exposure, as determined by a GWAS. The second and third assumptions are more difficult to satisfy. However, if the exposures are gene expression levels or protein levels, one can use *cis* genetic variants—which are variants found within a fixed distance (typically 500 kb or 1 mb) of the gene encoding the transcript or protein in question—to minimize pleiotropy and maximize biological plausibility. Additionally, directional pleiotropy can be tested using MR-Egger regression.^17^ A significant intercept term could indicate directional pleiotropy and a violation of the second assumption.

For our analysis, plasma protein levels were taken to be the exposure, and AAA was taken to be the outcome. AAA summary statistics were obtained from (GWAS) meta-analysis including 39,221 individuals with and 1,086,107 individuals without AAAs from 14 cohorts (AAAgen).^3^ The study comprised 37,214 individuals with AAAs from European populations and 2007 individuals with AAA from African populations. The individual studies comprising the meta-analysis relied on various International Classification of Diseases and phecode definitions of AAAs. Specific details can be found in the supplement of that meta-analysis. The summary statistics are available at https://csg.sph.umich.edu/willer/public/AAAgen2023/.

For exposure data, we used publicly available pQTLs identified in two large-scale plasma-proteomics studies (deCODE and UKB-PPP) to generate genetic instruments.^13^^,^^14^ The summary statistics for the deCODE dataset are available at https://www.decode.com/summarydata/, and for the UKB-PPP dataset at http://ukb-ppp.gwas.eu. Given that AAAgen relied, in part, on data from UKB and deCODE, we ran an identical MR analysis using only AAA summary statistics from the Million Veterans Program (MVP)—which, to our knowledge, has no overlap with the deCODE or UKB cohorts—as a sensitivity analysis to confirm our initial results.

Variants were chosen as instrumental variables if their nominal *P*-values achieved genome-wide significance (*P* < 5e-8). Only variants present in the exposure and outcome summary statistics were considered. The instrumental variables had effect sizes for plasma pQTL levels quantified in units of standard deviations, whereas the effect sizes for AAA were quantified in units of log-odds change in outcome, as originally reported in the summary statistics of their respective studies. To reduce the risk of pleiotropic effects, we only considered *cis* variants located within 500 kb of their respective genes. To select independent variants we performed linkage disequilibrium (LD) clumping using the *ld_clump* function from the ‘R’ package ‘ieugawsr’ (version 1.0.0).^18^ To allow more instruments per protein we relaxed the maximum LD R-squared threshold to 0.1. Otherwise, default argument values were used. To ensure consistent effect direction between studies, variant harmonization was performed using reference/alternate alleles included in the summary statistics and the *harmonise_data* function from the ‘R’ package ‘TwoSampleMR’ (version 0.5.9).^19^ When necessary, coordinates were converted from GRCh38 to GRCh37 coordinates using liftOver.^20^ Analyses were performed in GRCh37 coordinates. Overall, we were able to obtain instruments for 2783 total proteins between the two datasets.

We performed inverse-variance weighted MR using the *mr_ivw* function from the ‘R’ package ‘MendelianRandomization’ (version 0.10.0).^21^ In cases where there is only a single genetic variant per exposure, the *mr_ivw* function returns a Wald ratio. Due to our relaxed LD threshold for variants, we performed MR with the ‘correl’ argument set to ‘TRUE’ to allow for correlated instrumental variables. This adjusts the standard error of the instrumental variables to account for any residual correlation (LD) between them. The *mr_ivw* function returns effect size estimates for the effect of an exposure (plasma protein levels) on an outcome (AAAs). Effect sizes significantly above 0 or odds ratios (ORs) significantly above 1 indicate that increased plasma levels of a protein are associated with increased genetic liability for AAAs. The Benjamini-Hochberg procedure was used to calculate a false discovery rate (FDR). Results were deemed significant at a threshold of FDR <0.05 in each dataset. For all initially significant proteins with at least three instrumental variables, we performed MR-Egger using the ‘MendelianRandomization’ function *mr_egger*. For all initially significant proteins with at least two instrumental variables, we performed a leave-one-out analysis by iteratively leaving out each variant and performing MR as above. No power analyses were performed prior to our main analysis. We followed Strengthening the Reporting of Observational Studies in Epidemiology (STROBE)-MR guidelines in reporting our findings.^22^ The study protocol and details were not pre-registered.

### Initial colocalization analysis

We performed Bayesian colocalization between plasma protein levels and AAAs using the *coloc.abf* function from the ‘R’ package ‘coloc’ (version 5.2.3).^23^ This method calculates the posterior probabilities of four hypotheses: H0 (no causal variants for either trait), H1 (causal variant for the first trait but not the second), H2 (causal variant for the second trait but not the first), H3 (each trait has a distinct causal variant), and H4 (the two traits share a causal variant). In our case, the first trait was plasma protein levels of the protein of interest and the second trait was AAAs. When assessing colocalization between two traits, the hypothesis of interest is H4—with higher posterior probabilities providing stronger evidence of a shared causal variant. Default prior probabilities were used: *P*_1_ = 1e-4, *P*_2_ = 1e-4, *P*_12_ = 1e-5, where *P*_1_ and *P*_2_ are the prior probabilities that a variant is associated with trait 1 (plasma levels) and trait 2 (AAAs) respectively. *P*_12_ is the prior probability that the variant is associated with both traits. The default prior values for *P*_1_ and *P*_2_ are generally assumed to be widely applicable.^24^ For *P*_12_, we performed a sensitivity analysis using the *sensitivity* function from ‘coloc’ to get a range of *P*_12_ values for which H_4_ was greater than 0.5 and 0.7. We utilized all variants where summary statistics were available within 500 kb of genes encoding the proteins of interest. A posterior probability of a shared causal variant (H_4_) >0.7 was used as strong evidence of colocalization.

### Bidirectional MR

Bidirectional MR was performed to examine the possibility that genetic liability for AAAs was causal for changes in plasma protein levels. We used the same approach as in our initial MR analysis, this time using genetic liability for AAAs as the exposure and predicted plasma levels of our initially significant proteins as the outcome. However, all genome-wide significant (*P* < 5e-8) variants for AAAs were used as instruments (rather than only the significant *cis* variants used in the forward analysis).

### MR Steiger

In MR, instruments should ideally explain a larger proportion of the variance in the exposure than the outcome. Variants that explain more variance in the outcome than in the exposure may indicate poorly chosen instruments or suggest that the causal direction flows from outcome to exposure rather than vice versa. MR Steiger is an approach used to test this directionality hypothesis and to provide evidence for the true causal relationship between exposure and outcome. We performed MR Steiger using the *mr_steiger* function from the ‘R’ package ‘TwoSampleMR’ (version 0.5.9).^19^

### Gene ontology enrichment

Gene ontology (GO) enrichment analysis is a method used to identify which biological processes, molecular functions, or cellular components are overrepresented in a gene list of interest using Fisher’s exact test. GO analysis was performed using ‘Enrichr.’^25^ Our gene set of interest consisted of the 25 genes/proteins identified as significant in the MR analysis (FDR <0.05) that were also supported by colocalization (posterior probability H_4_ > 0.7). As a background, we used the set 2783 genes for which we were able to generate instruments and perform MR. Within our set of interest, we calculated the enrichment of ‘Reactome 2022’ pathways and ‘GO Cellular Component’ gene sets.

### Protein-protein interactions

Analysis was performed using ‘STRING.’^26^ STRING is a database containing experimentally demonstrated and computationally predicted protein-protein interactions (PPIs). One can use this database to determine if the number of PPIs observed in a gene set of interest are more numerous than expected by chance. For our analysis, the gene set of interest and the background were identical to the GO enrichment analysis. A limitation of STRING is that many of these PPIs have not been experimentally validated and may not represent true biological interactions. Furthermore, these PPIs are not phenotype or tissue specific: PPIs may be altered in different disease states and in different tissues.

### Gene expression

Median transcripts per million (TPM) values across tissues were obtained from GTEx Portal (GTEx v8).^27^ Expression levels were analyzed in vascular and endocrine tissues to account for plausible origins of plasma ECM proteins. For each of the eight ECM proteins, the median TPM levels within each tissue were Log_2_(TPM + 1) transformed. Those values were then divided by the Log_2_(TPM + 1) values in the tissue with the highest expression (of the 18 tissues analyzed).

### Colocalization of expression quantitative trait loci/splicing quantitative trait loci with pQTLs and AAAs

Bayesian multi-trait colocalization was performed using the *hyprcoloc* function from the ‘R’ package ‘HyPrColoc’ (version 1.0).^28^^,^^29^ Using summary statistics, this function allows one to calculate the posterior probability that all traits share a causal variant (similar to pp H_4_ in two-trait colocalization). We used default parameters and prior values. Re-analyzed GTEx aortic tissue splicing quantitative trait loci (sQTL) and expression quantitative trait loci (eQTL) summary statistics were obtained from ‘eQTL Catalogue.’^30^ All available variants within 500 kb of our genes of interest were used for the analysis.

## Results

### MR identifies 90 plasma proteins associated with AAA

We used two-sample MR to estimate the effects of genetically determined plasma protein levels on the genetic liability for AAA. We tested a total of 2783 unique plasma proteins, with 1701 from the deCODE dataset and 1957 from the UKB dataset, for associations with AAAs using two-sample MR. The number of genetic variants used as instruments per protein ranged from 1 to 197, with a median of 10 variants for the deCODE data and 15 variants for the UKB data (Supplementary Table 1).

**Fig 1:**
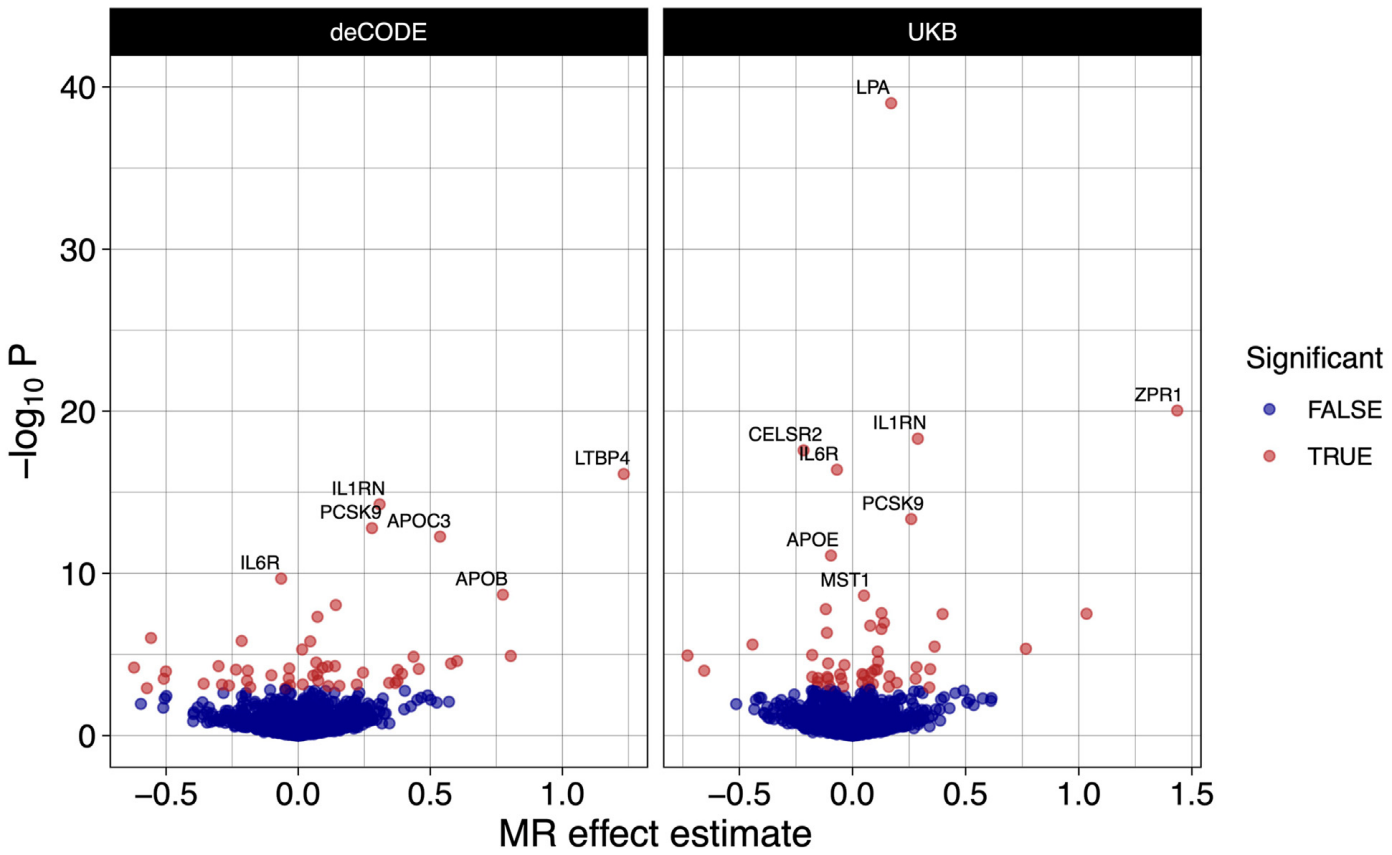
Mendelian randomization (*MR*) identifies 90 proteins associated with abdominal aortic aneurysms (AAAs). Instruments were generated and associations were tested for 2783 plasma proteins across the deCODE and UKB proteomics studies. The effect estimate is the natural logarithm of the odds ratio (OR). Significance was determined at false discovery rate (FDR) <0.05 in each dataset. Proteins with *P*-adj<1e-6 are labeled.

We found the genetically determined levels of 52 plasma proteins significantly associated with genetic liability to AAA (FDR <0.05) using instruments from the deCODE dataset and another 50 proteins associated with AAAs using instruments from the UKB dataset. (Fig 1; Supplementary Table II, online only). Overall, the genetically determined levels of 90 unique proteins were found to be significantly associated with genetic liability to AAAs. Of the 52 significant proteins found in the deCODE dataset, 23 were replicated in the UKB dataset (concordant direction of effect and nominal *P*-value < .05). Conversely, 17 of 50 proteins identified in the UKB dataset were replicated in the deCODE dataset (Supplementary Table III, online only). Notably, only 31 of 52 (60%) and 19 of 50 (38%) of the significant proteins found using instruments from the deCODE and UKB-PPP datasets respectively, had instruments in the other dataset. MR-Egger regression on plasma proteins significantly associated with AAAs found no evidence of substantial directional pleiotropy (Supplementary Table IV, online only). Leave-one-out analysis demonstrated that most significant proteins with multiple variants as instruments were not driven by a single variant (Supplementary Table V, online only). Only 15 of 77 initially significant proteins (with multiple single nucleotide polymorphisms [SNPs]) lost nominal significance (*P*-value > .05) when a single SNP was removed. None of the proteins showed a significant change in effect direction (ie, a significant negative effect did not change to a significant positive effect, or vice versa, when an SNP was dropped).

To further validate our initial findings, we conducted the same MR analysis using only AAA summary statistics from the MVP, because AAAgen (our original source of AAA summary statistics) partially incorporated data from UK Biobank (UKB) and deCODE (Supplementary Table VI, online only). The MVP cohort has no overlap with either the deCODE or UKB cohorts.

All proteins that were significant in our original analysis maintained their effect direction when using MVP statistics. Of the 52 significant proteins identified when using deCODE dataset as exposure and AAAgen as outcome, 45 maintained nominal *P*-values < .05 when using MVP summary statistics for the outcome (*P* < 1e-50, binomial test). Similarly, 47 of 50 initially significant proteins from the UKB dataset maintained nominal *P*-values < .05 with MVP summary statistics (*P* < 1e-56, binomial test). Given these consistent results, all downstream analyses were based on our initial MR analysis using AAAgen data for the outcome.

### Colocalization provides additional support for an association between plasma protein levels and AAAs

**Fig 2:**
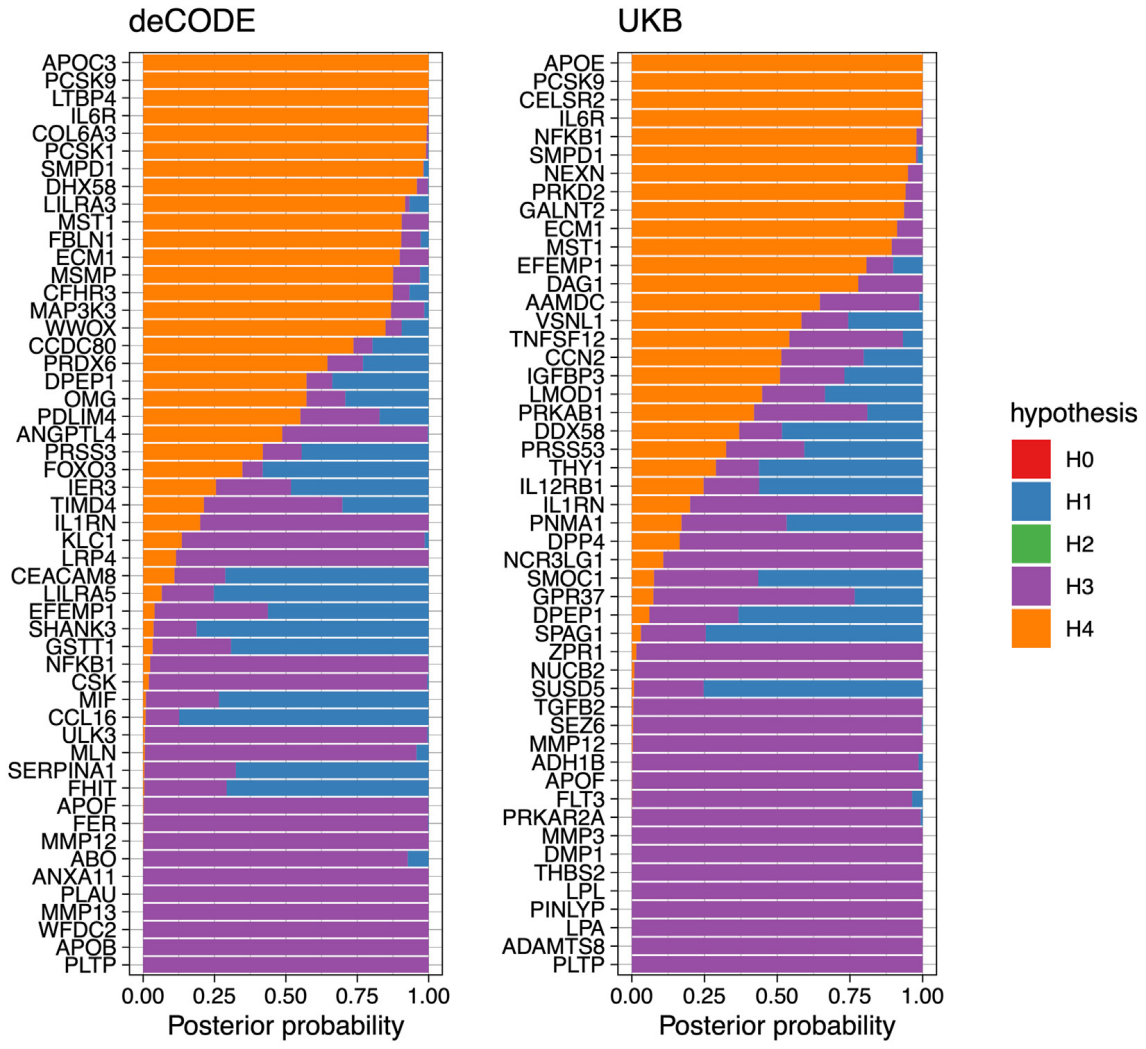
Colocalization analysis provides additional evidence for a causal association between protein levels and abdominal aortic aneurysms (AAAs). Bayesian colocalization results for all plasma proteins with significant associations with AAA (false discovery rate [FDR] <0.05). The hypotheses are as follows: H0, no causal variant for either trait; H1, causal variant for plasma levels only; H2, causal variant for AAA only; H3, two distinct causal variants; and H4, one shared causal variant for plasma levels and AAAs. Twenty-five distinct plasma proteins had posterior probability for H4 >0.7, showing that the plasma protein levels and AAAs likely share causal variants.

To provide additional support for the association between genetically determined levels of plasma proteins and genetic liability, we tested for evidence of colocalization between pQTL and their cognate AAA risk loci.^31^ This approach can be helpful for distinguishing putative causal associations from those confounded by linkage disequilibrium.^32^ We used all *cis* variants within 500 kb of their respective genes to perform colocalization. For 25 of the 90 proteins (27%) whose genetically determined plasma levels associated with genetic liability to AAAs, there was evidence of colocalization (posterior probability H4 >0.7), indicating that the plasma levels of these proteins and AAA likely share the same causal variants (Fig 2; Supplementary Table VII, online only). Among those supported by both MR and colocalization were previously experimentally validated proteins such as PCSK9 (OR, 1.3; 95% confidence interval [CI], 1.2-1.4; *P* < 1e-10) and other proteins, including LTBP4 (OR, 3.4; 95% CI, 2.6-4.6; *P* < 1e-10) and COL6A3 (OR, 0.6; 95% CI, 0.5-0.7; *P* < 1e-6).^3^

### AAA-associated proteins are enriched for proteins found in ECM

**Fig 3:**
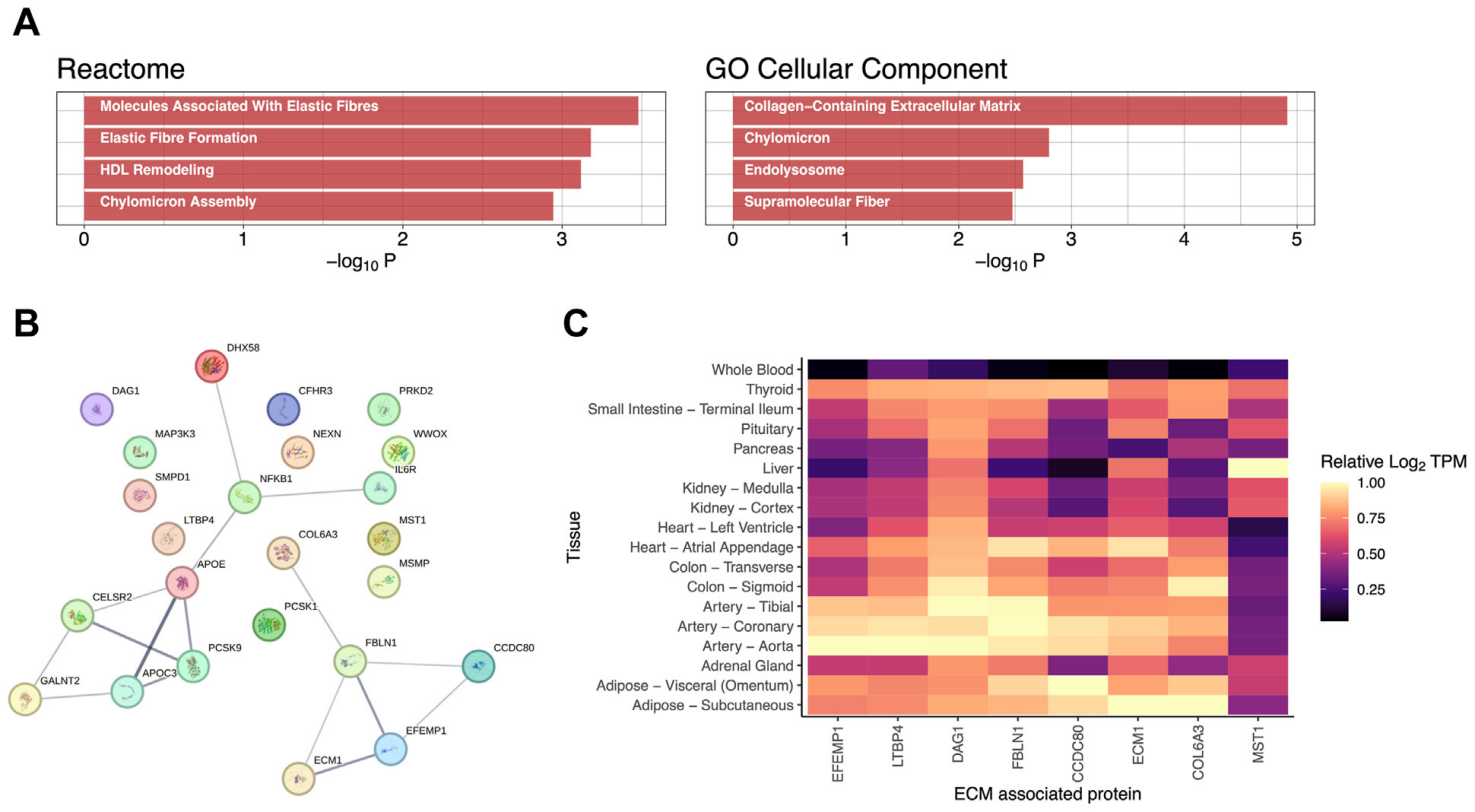
Biological properties of proteins associated with abdominal aortic aneurysms (AAAs). **A**, The 25 proteins supported by Mendelian randomization (MR) and colocalization were tested for significant overrepresentation of pathways and gene ontology (*GO*) terms. The most significant reactome pathway was “Molecules Associated with Elastic Fibers” (odds ratio [OR], 29; *P* < 1e-3), whereas the most significant GO cellular component term was “Collagen-Containing Extracellular Matrix” (OR, 7.8; *P* < 1e-4). All terms and pathways shown were significant at false discovery rate [FDR] <0.05. **B**, Protein-protein interactions (PPIs) among proteins supported by both MR and colocalization. The PPIs were identified in the STRING database. Increasing edge thickness indicates increasing strength of supporting evidence for PPI. **C**, The expression levels of extracellular matrix (*ECM*)-associated proteins identified by MR and colocalization across tissues. Expression levels are shown relative to expression levels in the tissue with highest expression (among tissues shown). Median mRNA levels of the genes (transcripts per million [TPM]) in each tissue were Log_2_(TPM + 1) transformed and divided by the Log_2_(TPM + 1) transformed median TPM of the tissue with maximal expression. A value of 1 indicates that the tissue has the highest expression level for that gene of the 18 tissues analyzed. Several of the ECM proteins show some of their highest expression levels in aortic tissue.

Among plasma proteins supported by both MR and colocalization, GO enrichment analysis of reactome pathways found an overrepresentation of proteins associated with elastic fibers (OR, 29; *P* < 1e-3), elastic fiber formation (OR, 22; *P* < 1e-3), and high-density lipoprotein remodeling (OR, 80; *P* < 1e-3) (Fig 3, *A*; Supplementary Table VIII, online only).^33^ GO analysis of cellular components revealed an enrichment of proteins found in collagen-containing ECM (OR, 7.8; *P* < 1e-4), chylomicrons (OR, 48; *P* = 1.6e-3), and endolysosomes (OR, 34; *P* = 2.7e-3) (Fig 3, *A*; Supplementary Table VIII, online only). Although our results were consistent with the current understanding of AAAs as a disease influenced by ECM and lipid metabolism dysregulation,^8^ the fact that the ECM signal was generated from QTL derived from plasma measurement of proteins was not expected. To gain additional insights into the biology of these proteins, we investigated their PPIs (Fig 3, *B*). Consistent with our GO analysis, we found that two clusters emerged: a cluster consisting of proteins involved in lipid metabolism and inflammation and another cluster consisting of ECM proteins. The 24 genes included in this analysis (PPI data could not be found for LILRA3) had 16 known interactions among them, which was significantly higher than expected by chance (expected = 9; *P* = .015).

To help determine the origin of the AAA-associated ECM proteins we identified in blood plasma, we examined gene expression data from endocrine and blood-adjacent tissues found in the Genotype-Tissue Expression Project (GTEx).^27^ We found that the proteins LTBP4, FBLN1, EFEMP1, and DAG1 had their highest mRNA levels in aortic or arterial tissues (Fig 3, *C*). Additionally, CCDC80 and ECM1 had some of their highest expression levels in aortic tissue. These results indicate that the AAA-associated ECM proteins we identified in plasma plausibly originated in the aortic wall.

### Bidirectional MR shows that AAA is not likely to be driving the changes in plasma levels of ECM proteins

To examine the possibility that AAA was causing the levels of ECM proteins to change in blood plasma, we performed a second MR experiment, this time with genetic liability to AAAs acting as the exposure and genetically predicted plasma levels of each protein as the outcome (bidirectional MR). We performed bidirectional MR on all 90 proteins that we prioritized in our initial MR analysis. After accounting for multiple testing, only 6.7% (6/90) were putatively affected by genetic liability to AAAs (FDR <0.05) (Supplementary Table IX, online only). Notably, none of the ECM proteins appeared to have their circulating levels influenced by genetic susceptibility to AAAs.

As a complementary analysis, we used MR Steiger, which allows one to test whether an instrumental variable is more strongly associated with the exposure than the outcome—indicating that the exposure likely caused the outcome and not the reverse. We found that every instrumental variable used for proteins significantly associated with AAA had the appropriate directionality (Supplementary Table X, online only), further showing that AAAs were unlikely to cause the observed changes in plasma protein levels.

### Variants affecting the expression and splicing of ECM proteins in the aortic wall may be driving the association with AAAs

**Fig 4:**
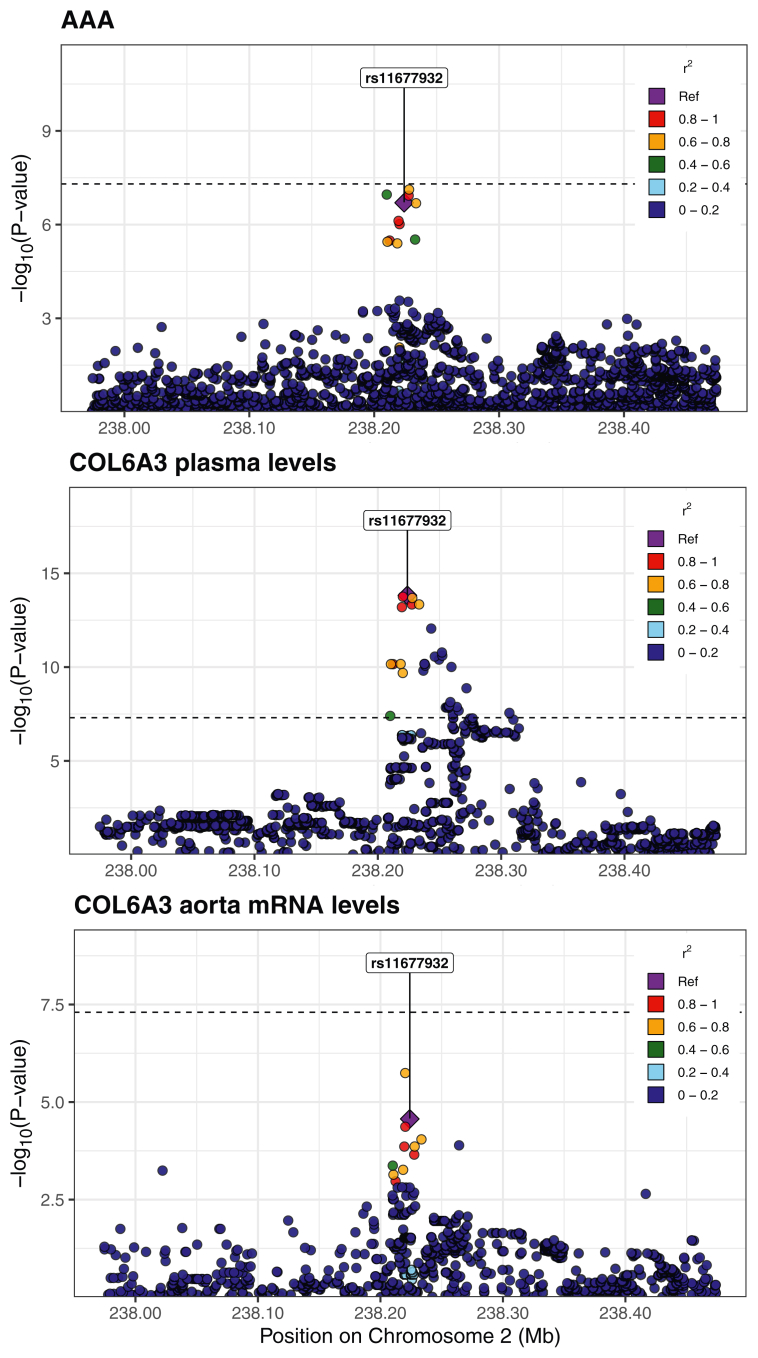
Regional association plots for COL6A3. Plots demonstrating the colocalization between variants associated with mRNA levels of COL6A3 in aorta, plasma levels of COL6A3, and abdominal aortic aneurysms (*AAAs*). The posterior probability for the colocalization of all three traits is 0.84.

To gain insight into the biological mechanism by which genetic variation may lead to changes in ECM proteins in the aorta, and subsequently changes in plasma levels of these proteins, we examined eQTLs and sQTLs in aortic tissue from GTEx. We performed a Bayesian colocalization analysis to determine whether the variants altering gene expression (mRNA levels or splicing) were also the same variants leading to changes in plasma levels and AAAs. We considered all ECM proteins with expression TPM >10 in aortic tissue. We found that the eQTL for the ECM protein COL6A3 colocalized with its plasma pQTL and AAA with a posterior probability of 0.84 (Fig 4). At the putative causal variant, as determined by HyPrColoc,^28^^,^^29^ (GRCh38, chr2:237,315,312 A/G; rs11677932) the allele associated with increased aortic expression (G), was also associated with increased plasma levels and decreased risk of AAAs.

The aptamer (used to measure plasma levels) for COL6A3 binds to the C-terminus, which is cleaved to a peptide called endotrophin.^34^, ^35^, ^36^ Given that COL6A3 had the highest expression levels in subcutaneous adipose tissue (in GTEx), and that plasma endotrophin has been linked to adipose tissue, we examined the possibility that the plasma COL6A3 originated in adipose tissue rather than aortic tissue. Hence, we performed an eQTL-pQTL-AAA colocalization analysis using eQTL data from subcutaneous adipose tissue. We found that the three signals did not colocalize (posterior probability = 6e-4), further adding evidence that increased plasma levels of COL6A3/endotrophin are caused by expression changes in aortic tissue.

**Fig 5:**
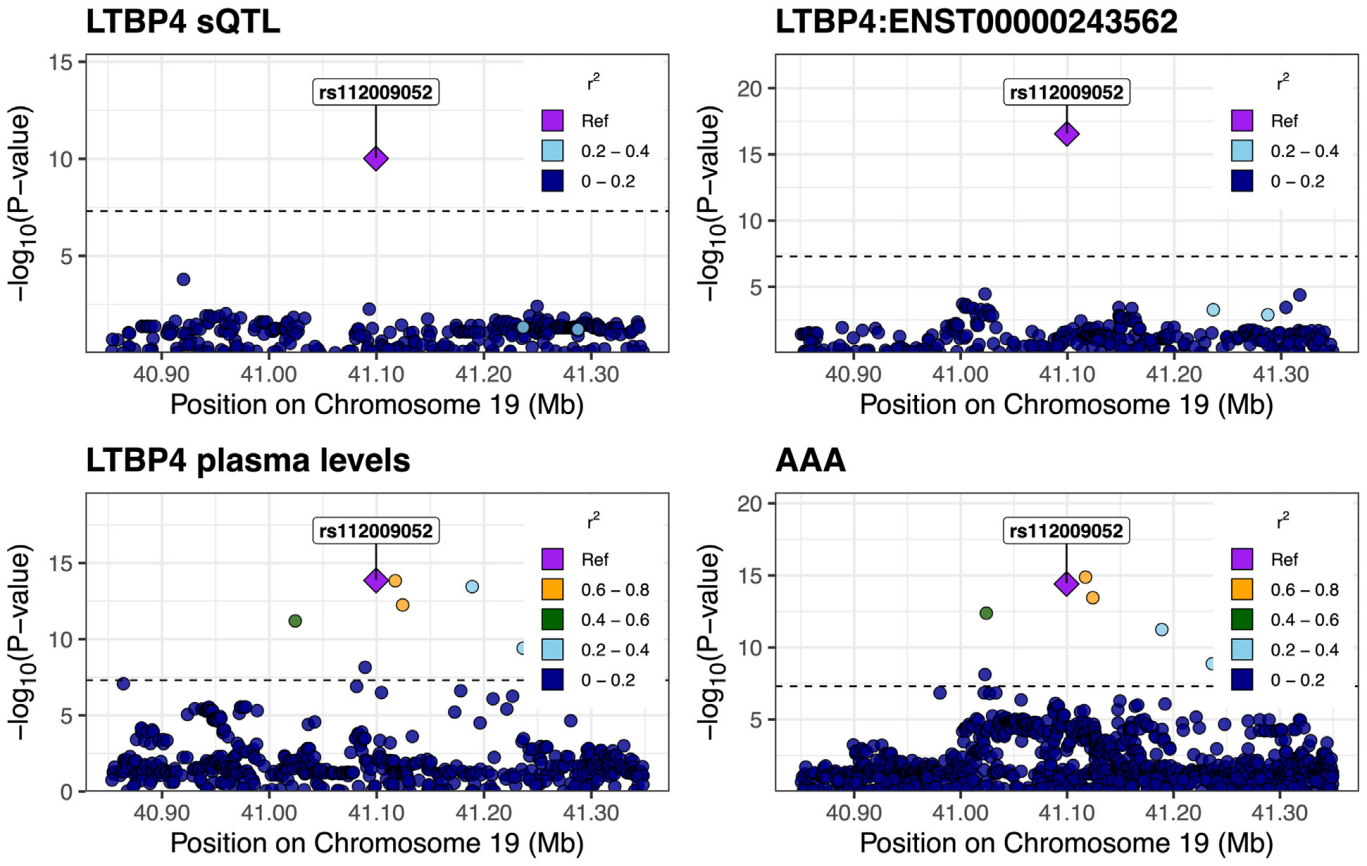
Regional association plots for LTBP4. Regional association plots demonstrating colocalization of splicing quantitative trait locus (*sQTL*) and protein quantitative trait loci (pQTL) variants for LTBP4, with variants associated with abdominal aortic aneurysms (*AAAs*). The posterior probability for the colocalization of all four traits is 0.89.

We then examined sQTLs, and found that for LTBP4, at the putative causal variant (GRCh38, chr19: 40,593,595 A/T; rs112009052), the allele (T) associated with increased splicing out of an intron (GRCh38 chr19: 40,611,394:40,611,859) was associated with decreased transcription of a corresponding truncated transcript that does not use that splice junction pair (ENST00000243562). This allele was also associated with increased LTBP4 plasma levels and increased liability for AAAs. The posterior probability for the colocalization of those four traits (sQTL, transcript-level-QTL, pQTL, and AAA) was 0.89 (Fig 5).

To further investigate whether the expression or splicing changes cause changes in circulating protein levels, we performed MR. We used the putative causal variants identified by HyPrColoc as instruments, with eQTL (for COL6A3) or sQTL (for LTBP4) data as the exposure and pQTL data as the outcome. Our analysis revealed that COL6A3 aortic expression levels were significantly associated with COL6A3 plasma levels (OR, 1.37; 95% CI, 1.26-1.48, *P* = 1.5e-14; Wald ratio). Similarly, LTBP4 splicing in the aorta was significantly associated with LTBP4 plasma levels (OR, 1.15; 95% CI, 1.11-1.20; *P* = 1.4e-14; Wald ratio). These findings support the hypothesis that changes in expression or splicing in the aortic wall may drive alterations in plasma protein levels.

## Discussion

We performed proteome-wide MR to identify plasma proteins that are potentially causal in the development of AAAs. Our analysis identified 90 distinct proteins where the genetically determined levels of the protein were associated with genetic liability to AAAs. Of those, 25 were supported by colocalization analysis, indicating that the circulating protein levels and AAAs shared causal variants. Unexpectedly, we found that the putatively causal proteins were enriched for proteins typically found in ECM, with several of the ECM proteins having their highest expression levels in aortic tissue.

Prior research has indicated that increased expression of matrix metalloproteinases directly leads to aneurysmal growth in AAAs.^37^ Matrix metalloproteinases are thought to induce aneurysmal growth through the breakdown of ECM proteins and thus the weakening of the aortic wall. Given this relationship, we investigated whether genetic liability for AAAs might be causal for increased levels of circulating ECM proteins. However, our bidirectional MR and MR Steiger analyses demonstrated a lack of evidence for that hypothesis.

To examine the relationship between the aortic expression and the circulating levels of ECM proteins, we performed a colocalization analysis. We sought to see if the variants responsible for changes in mRNA levels were also causal for changes in plasma levels and AAAs. We examined all ECM proteins expressed in the aorta, and found that the eQTL colocalized (posterior probability = 0.84) with the pQTL and AAA variants for the protein COL6A3. All other eQTL-pQTL-AAA colocalization probabilities were below 0.5 for ECM proteins. We found additional support for the hypothesis that increased COL6A3 expression in the aorta causes an increase in COL6A3 plasma levels using MR.

The aptamer used to measure levels of COL6A3 in the deCODE proteomics dataset binds the C-terminal of COL6A3.^13^ The C-terminal of COL6A3 gets cleaved into a peptide known as endotrophin, which can be found in the bloodstream.^34^^,^^35^ Although expression data suggests that endotrophin may largely originate from subcutaneous adipose tissue, our colocalization analysis supports an alternative hypothesis: plasma endotrophin may primarily originate in aortic or vascular tissue.

Interestingly, our findings on endotrophin’s relationship with AAAs contrast with its associations with other cardiovascular conditions. Clinical, functional, and computational studies have linked endotrophin to inflammation, insulin resistance, coronary artery disease, and heart failure.^34^, ^35^, ^36^ Notably, increased circulating levels of endotrophin were associated with increased risk of coronary artery disease and heart failure. However, our results suggest that increased plasma endotrophin may be protective against AAAs. We hypothesize that this apparent protective effect against AAAs might be related to endotrophin-induced insulin resistance, as insulin resistance has previously been associated with reduced rates of AAAs.^38^ However, this relationship requires further investigation to establish causality and understand the underlying mechanisms.

To gain further insight into whether expression-level changes in the aortic wall were possibly responsible for AAA, we performed colocalization analysis between sQTLs, pQTLs, and AAAs. Among all ECM proteins expression in aortic tissue, we found that a sQTL for LTBP4 colocalized with its plasma pQTL and a significant locus for AAAs. Furthermore, MR analysis provided additional evidence supporting the hypothesis that increased LTBP4 splicing in the aorta leads to an increase in LTBP4 plasma levels. These results suggest that splicing alterations in LTBP4 in the aortic wall may affect its plasma levels and AAAs. Notably, across tissues, we found that LTBP4 is most highly expressed in aortic tissue (Fig 3, *C*).

There are several different causal pathways by which ECM proteins, which are primarily expressed in the abdominal aortic wall, can lead to AAAs while changing their circulating levels. The changes in the plasma levels themselves may or may not be causal in the formation of AAAs. In the case that the plasma levels are not directly causal, and assuming that their origin is indeed the aorta, we can regard the plasma levels of these proteins as quantitative proxies for their ‘dysfunction’ within the aortic wall without invalidating the assumptions of MR. Prior research has demonstrated that LTBP4 is involved in the formation of elastic fibers,^39^^,^^40^ which is crucial to the integrity of the aorta.^37^ Therefore, it seems plausible that the original lesion in the causal pathway occurs in the aortic wall, and the elevated plasma levels of LTBP4 simply reflect its dysfunction in the aorta.

**Fig 6:**
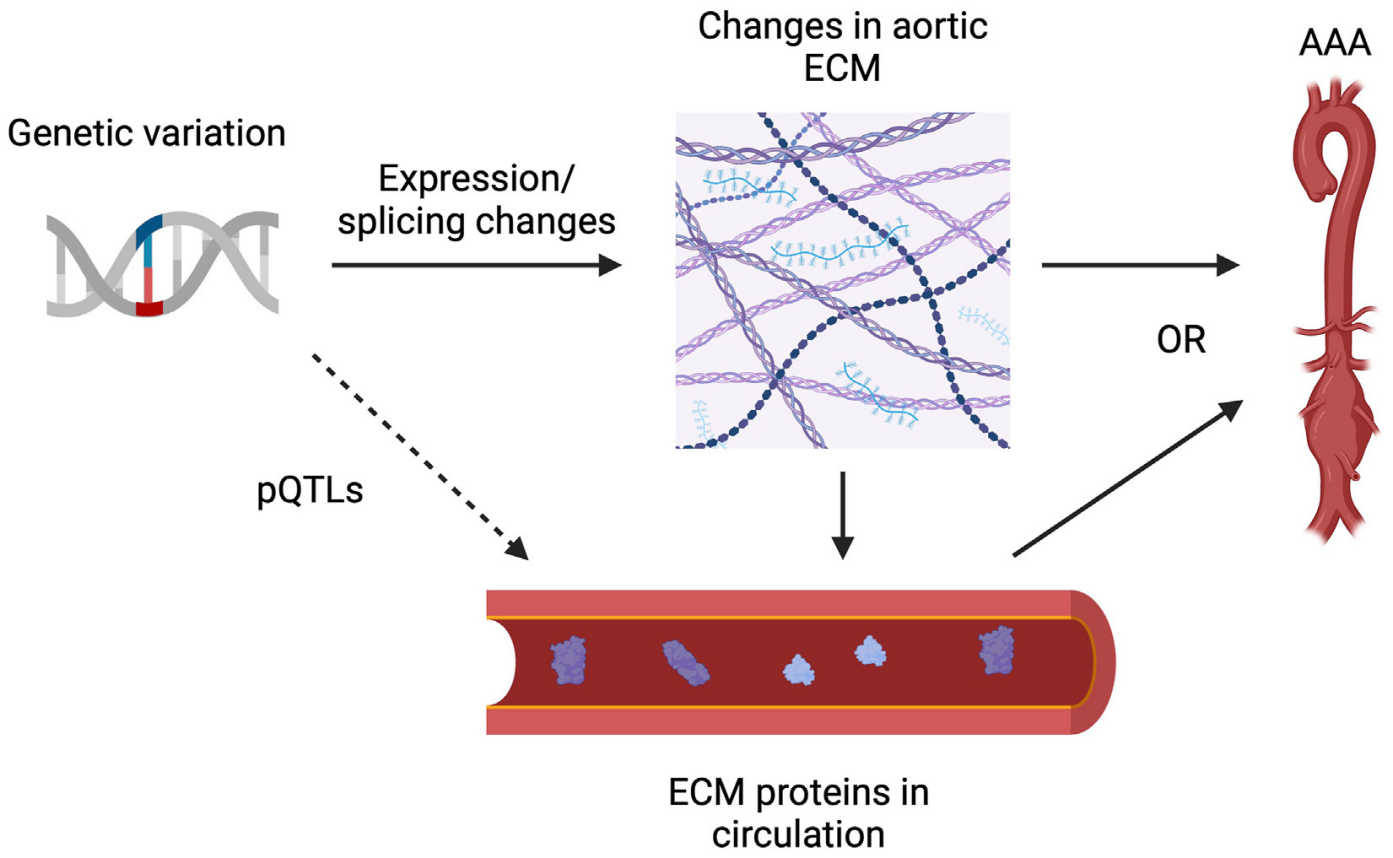
A causal model for abdominal aortic aneurysms (*AAAs*) through extracellular matrix (*ECM*) proteins. Genetic variation alters the expression or splicing of ECM proteins, which leads to changes in the aortic wall. These aortic ECM changes lead to AAAs, while simultaneously causing ECM proteins to leak out into the aortic lumen. Alternatively, the ECM proteins in circulation themselves cause AAA, rather than the aortic ECM changes. Both possibilities, or a combination of the two, are consistent with our findings and the assumptions of Mendelian randomization (MR). *pQTL*, Protein quantitative trait loci. This figure was generated using BioRender.

Given our findings, we propose a model for how ECM proteins found in plasma can cause AAAs (Fig 6). Our findings suggest two plausible mechanisms linking genetic variation to AAAs. In one scenario, genetic factors alter the expression or splicing of ECM proteins, leading to changes in the aortic wall. These alterations may directly contribute to AAA development while simultaneously causing ECM proteins to leak into the aortic lumen. Alternatively, the circulating ECM proteins themselves may be causative agents for AAAs, independent of aortic wall changes. It's also possible that a combination of both mechanisms is at play. Importantly, both hypotheses align with our findings and adhere to the assumptions of MR. Based on biological plausibility, the first hypothesis appears to be the most likely. However, to assess the relative importance of the second hypothesis, future experiments could potentially inject purified ECM proteins or peptides into rodent models to see if this alters AAA development.

This study has several limitations despite our findings. First, not all assumptions underlying MR can be definitively proven. Although we attempted to minimize the risk of violation (see Methods), two key assumptions remain unprovable: (1) that the IVs are associated with the outcome (AAA) only through the exposure (circulating protein levels); and (2) that the IVs are not associated with confounders affecting both the exposure and outcome. Additional experimental evidence is therefore necessary to prove causality.

A second limitation concerns our bidirectional MR analysis, which relied on both *trans*-pQTLs and *cis*-pQTLs to rule out the possibility that genetic liability for AAAs causes changes in plasma ECM protein levels. However, the datasets used to discover these pQTLs are likely underpowered to detect trans-pQTLs, potentially affecting the robustness of this analysis. Another limitation concerns the use and interpretation of pQTL data: the aptamers and antibodies used to quantify plasma protein levels bind isoforms of the same gene with different affinities. This makes the interpretation of pQTL data challenging when there are meaningful biological differences between the isoforms of a given gene. Finally, given that this study primarily utilized data from individuals of European ancestry, further research across diverse populations is necessary to assess the generalizability of these findings.

## Conclusions

Overall, our results highlight proteins and pathways with potential causal effects on AAAs, providing a foundation for future functional experiments. These findings suggest a possible causal model for the formation of AAAs through ECM proteins found in plasma. If supported by additional evidence, future medical therapies for AAAs may focus on altering the expression patterns of ECM proteins in the aortic wall.

## Author Contributions

Conception and design: SK, ML, SD

Analysis and interpretation: SK, SY, JS, PT, ML, SD

Data collection: Not applicable

Writing the article: SK, SY, ML, SD

Critical revision of the article: SK, SY, JS, PT, ML, SD

Final approval of the article: SK, SY, JS, PT, ML, SD

Statistical analysis: SK

Obtained funding: Not applicable

Overall responsibility: ML and SD

ML and SD contributed equally to this article and share co-senior authorship.

## Funding

This work was supported by the National Institutes of Health National Heart, Lung, and Blood Institute R01HL166991. M.G.L. was supported by the Doris Duke Foundation (Award 2023-0224) and the United States Department of Veterans Affairs Biomedical Research and Development Award IK2-BX006551.

## Disclosures

S.M.D. receives research support to the University of Pennsylvania from RenalytixAI and in-kind support from Novo Nordisk, and is a paid consultant for Tourmaline Bio. M.G.L. receives research support to the University of Pennsylvania from MyOme, and consulting fees from BridgeBio, both outside the scope of this research. The content of this manuscript does not represent the views of the Department of Veterans Affairs or the United States Government.

## Appendix (online only)

Supplementary Table Descriptions
